# Thiosemicarbazide Derivatives Targeting Human TopoIIα and IDO-1 as Small-Molecule Drug Candidates for Breast Cancer Treatment

**DOI:** 10.3390/ijms24065812

**Published:** 2023-03-18

**Authors:** Barbara Kaproń, Robert Czarnomysy, Dominika Radomska, Krzysztof Bielawski, Tomasz Plech

**Affiliations:** 1 Department of Clinical Genetics, Medical University of Lublin, Radziwiłłowska 11, 20-080 Lublin, Poland; 2 Department of Synthesis and Technology of Drugs, Medical University of Bialystok, Kilińskiego 1, 15-089 Białystok, Poland; 3 Department of Pharmacology, Medical University of Lublin, Radziwiłłowska 11, 20-080 Lublin, Poland

**Keywords:** breast cancer, thiosemicarbazide derivatives, dual-targeting agents, flow cytometry, ADME-Tox

## Abstract

In 2020, breast cancer became the most frequently diagnosed type of cancer, with nearly 2.3 million new cases diagnosed. However, with early diagnosis and proper treatment, breast cancer has a good prognosis. Here, we investigated the effect of thiosemicarbazide derivatives, previously identified as dual inhibitors targeting topoisomerase IIα and indoleamine-2,3-dioxygenase 1 (IDO 1), on two distinct types of breast cancer cells (MCF-7 and MDA-MB-231). The investigated compounds (**1**–**3**) selectively suppressed the growth of breast cancer cells and promoted apoptosis via caspase-8- and caspase-9-related pathways. Moreover, these compounds caused S-phase cell cycle arrest and dose-dependently inhibited the activity of ATP-binding cassette transporters (MDR1, MRP1/2 and BCRP) in MCF-7 and MDA-MB-231 cells. Additionally, following incubation with compound 1, an increased number of autophagic cells within both types of the investigated breast cancer cells was observed. During preliminary testing of ADME-Tox properties, the possible hemolytic activities of compounds **1**–**3** and their effects on specific cytochrome P450 enzymes were evaluated.

## 1. Introduction

Currently, cancer is recognized as a leading cause of death worldwide, being responsible for 10 million deaths in 2020 [1]. Additionally, in the same year, breast cancer (BC) became the most frequently diagnosed type of cancer, with nearly 2.3 million new cases. However, with early diagnosis and proper treatment, BC has a good prognosis. One of the most widely recognized classifications of BC is based on the immunohistochemical expression of estrogen receptors (ER), progesterone receptors (PR) and human epidermal growth factor receptors (HER2). Among the four classified subtypes of BC, luminal A and luminal B subtypes are ER and/or PR positive and the HER2 subtype is characterized by high HER2 expression and a lack of ER/PR, while triple-negative BC (TNBC) is characterized by the absence of ER/PR and HER2 overexpression [2]. Depending on the type and grade of BC, its treatment includes surgery, radiation and systemic therapy (chemotherapy, hormone therapy, immunotherapy and targeted biological therapy). Pharmacological treatment can be applied before or after the surgical intervention, i.e., as neoadjuvant or adjuvant therapy, respectively. The main objective of chemotherapy in BC is to destroy cancer cells and to reduce their ability to spread from the primary tumor to other parts of the body. Traditional cytotoxic agents still remain an important part of therapy in BC; however, their efficacy is seriously confined by their low selectivity and dose-limiting toxic effects against human normal cells. Since one of the objectives of the WHO, in relation to cancer policy, is to reduce BC mortality by 2.5% annually, there is a vital need to improve the diagnosis and treatment of patients suffering from BC [3]. 

**Figure 1:**
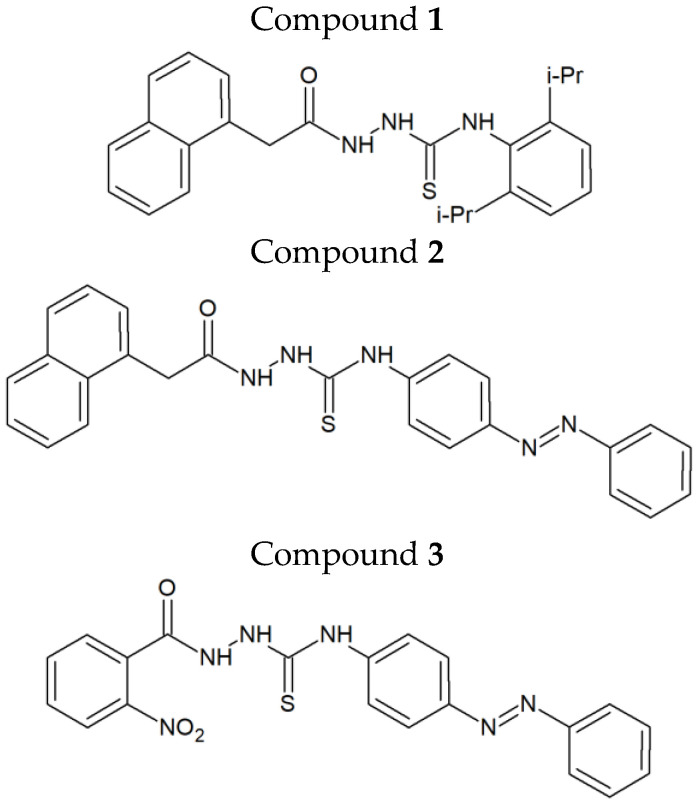
First in-class dual inhibitors targeting human topoisomerase IIα and indoleamine-2,3-dioxygenase 1.

In our recently published work, we identified the firstin-class dual inhibitors targeting both human DNA topoisomerase IIα and indoleamine-2,3-dioxygenase 1 (IDO 1) (Figure 1) [4]. These compounds exerted strong cytotoxic and antiproliferative activity, up to 90times higher than that of etoposide, in various human cancer cell lines. Among the two breast cancer cell types (MCF-7 and MDA-MB-231) investigated during our preliminary studies, MCF-7 cells turned out be more sensitive to the above-mentioned agents. Compound **1**, when examined against MCF-7 cells in aBrdU assay, showed a 46times stronger antiproliferative activity than etoposide and was much more selective against cancer cells than normal (non-cancerous) cells, as indicated by its selectivity index (SI = 46.8). As shown by the example of etoposide, topoisomerase IIα inhibitors can be important elements ofBC treatment. Due to the relatively low toxicity and beneficial clinical response, orally administered etoposide is regarded as a safe option for patients with heavily pre-treated metastatic breast cancer, especially for those not responding to other medical therapies [5,6]. Bearing in mind that compounds **1**–**3** (Figure 1) possess additional IDO 1 inhibitory activity, they may constitute an interesting option in cancer treatment. It is known from the literature that co-administration of IDO 1 inhibitors with radiotherapy or systemic therapy improves the results of the treatment [7]. IDO 1 inhibitors have also been comprehensively examined in clinical trials as potentially useful drugs to overcome tumor-induced immunosuppression [8]. 

Both topoIIα and IDO 1 were reported to be highly upregulated in breast cancer patients [9,10]. The measurement of topoIIα in invasive BC has an important clinical value, since it gives information on the quantity of cycling tumor cells [11]. Additionally, significant correlation was observed between HER-2/neu oncoprotein overexpression and the increased topoIIα level in breast tumors [9]. The increased expression of topoIIα was observed in higher stage tumors [12]. Likewise, IDO overexpression is frequent among high-grade TNBC specimens [13]. High levels of IDO 1 within BC cells are also correlated with microvessel density and a worse clinical prognosis [14]. Therefore, there is a strong rationale for the use of topoIIα/IDO 1 inhibitors as possible anti-BC agents.

In the present study, we aimed to investigate the effects of thiosemicarbazide derivatives 1–3 on the mechanisms affecting breast cancer cells functions, including their different effects on ER/PR positive cells (MCF-7) and TNBC cells (MDA-MB-231). Moreover, an in vitro screening of some ADME-Tox properties of compounds 1–3 was carried out in order to better characterize them as possible drug candidates.

## 2. Results

### 2.1. Preparation of the Investigated Compounds

Thiosemicarbazide derivatives 1–3 were prepared via reaction of the carboxylic acid hydrazides and isothiocyanates in one-step reaction performed in boiling ethanol. Their structures were designed using in silico methods and are presented in Figure 1. The design, synthesis and spectral characterization of the compounds have been described in our previously published paper [4].

### 2.2. Viability of Human Normal Breast Epithelial Cells (MCF-10A) Exposed to Compounds ***1**–**3*** for 24 h

The cytotoxic effect of compounds **1**–**3** against MCF-7 and MDA-MB-231 cells was evaluated previously and described in [4]. However, the selectivity of newly designed anticancer agents towards cancer cells compared to normal cells is crucial to develop new treatment options. In order to examine the selectivity of the tested compounds towards MCF-7 and MDA-MB-231 breast cancer cells, the viability of human normal breast epithelial cells (MCF-10A) incubated for 24 h with increasing concentrations of compounds **1**–**3** was determined using MTT assays. The median inhibitory concentrations (IC_50_) of compounds **1**–**3** were 25.31 ± 2.27, 42.74 ± 3.16 and 40.45 ± 4.59 µg/mL, respectively (Table 1). In turn, the values of the selectivity index (SI) ranged from 2.57 to 5.30 for MCF-7 and from 2.75 to 5.60 for MDA-MB-231. Human normal MCF-10A cells were less sensitive to thiosemicarbazide derivatives 2 and 3 when compared to compound **1**.

### 2.3. Effect of Compounds ***1**–**3*** on the Apoptosis in MCF-7 and MDA-MB-231 Cells

**Figure 2:**
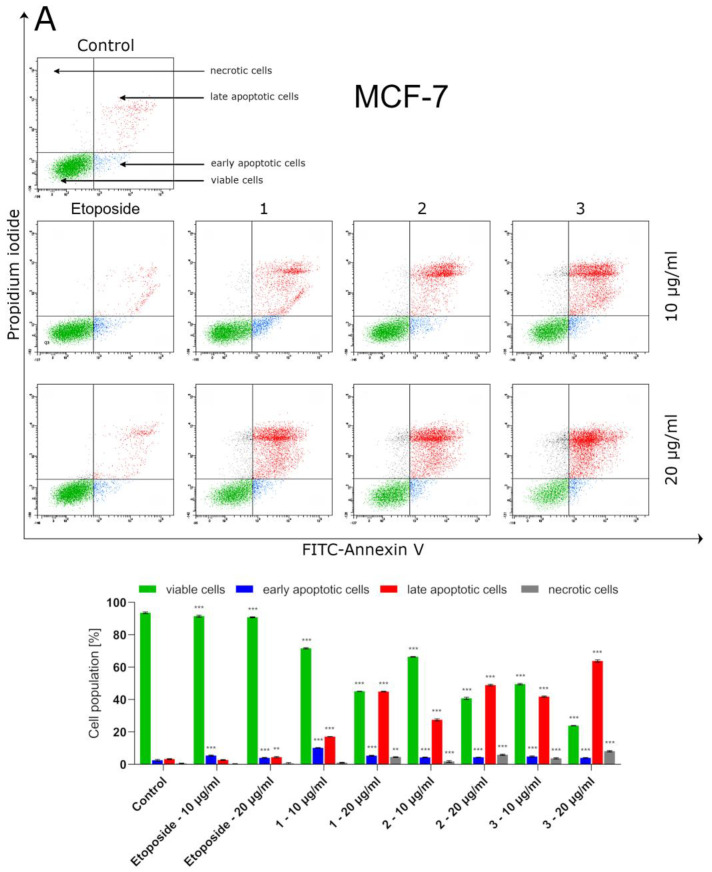

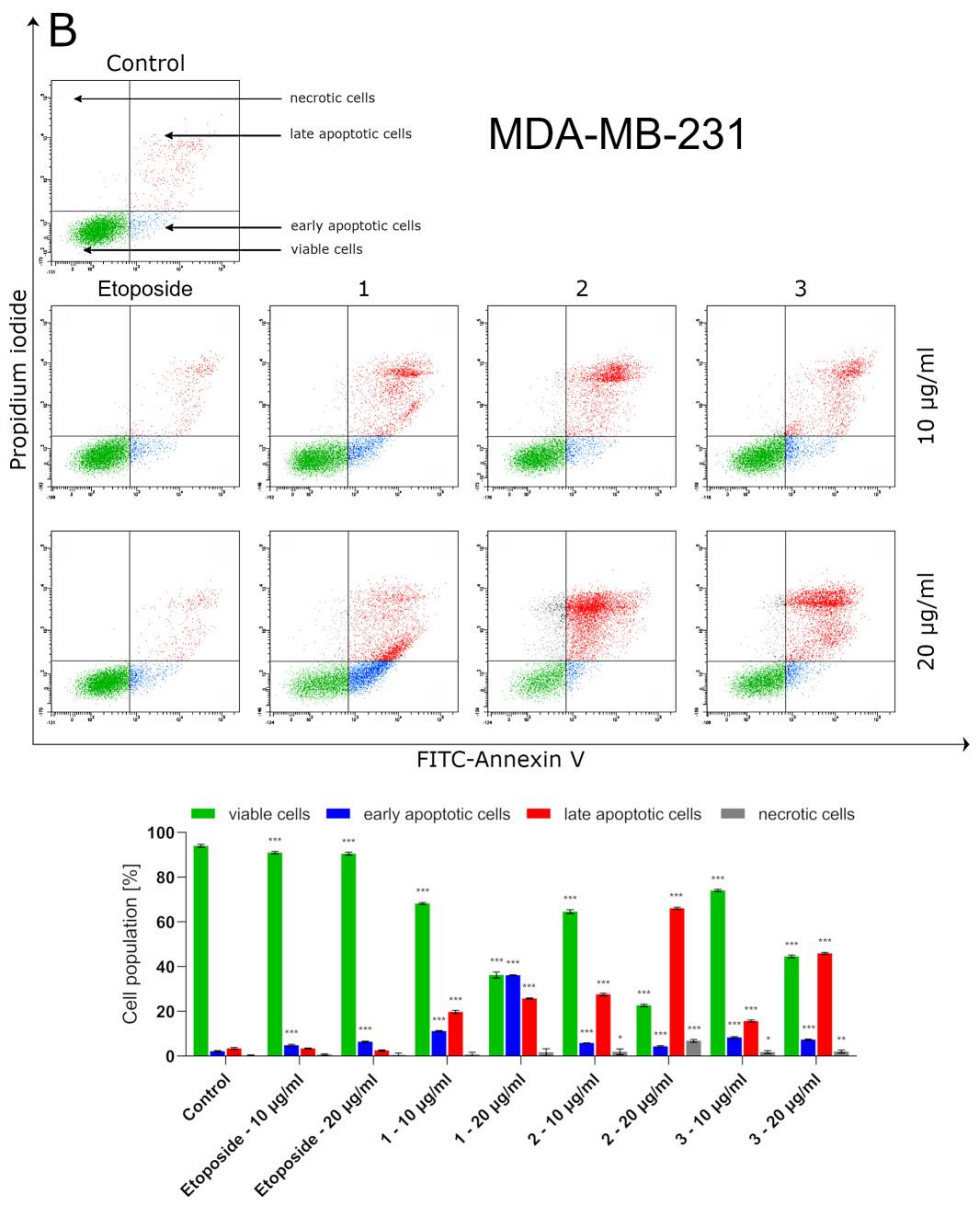
Flow cytometry analysis of MCF-7 (**A**) and MDA-MB-231 (**B**) breast cancer cells after 24 h incubation with compounds **1**–**3** and etoposide, and subsequent staining with Annexin V and propidium iodide. The compounds were tested at the following concentrations: **1** (10 µg/mL/23.83 µM and 20 µg/mL/47.66 µM), **2** (10 µg/mL/22.75 µM and 20 µg/mL/45.50 µM), **3** (10 µg/mL/23.78 µM and 20 µg/mL/47.56 µM) andetoposide (10 µg/mL/17 µM and 20 µg/mL/34 µM). Mean percentage values from three independent experiments (n = 3) performedin duplicate are presented. * *p* < 0.05 vs. control group,** *p*< 0.01 vs. control group, *** *p* < 0.001 vs. control group.

The investigated thiosemicarbazide derivatives (1–3) induced apoptosis in MCF-7 and MDA-MB-231 cells in a dose-dependent manner (Figure 2). The pro-apoptotic activities of 1–3 were much higher than that of etoposide. It is worth mentioning that the ability of the tested compounds to induce apoptosis in breast cancer cells was correlated with their cytotoxic properties observed in MTT assays. Compound **3**, which induced apoptosis in nearly 68% of MCF-7 cells, also most potently impaired their viability, with an IC_50_ of 7.67 µg/mL. Similarly, MDA-MB-231 cells were most sensitive to derivative 2 (IC_50_= 7.64 µg/mL), which caused the greatest increase in the number of apoptotic cells to 70%. At the same time, MCF-7 and MDA-MB-231 cells incubated with etoposide (20 µg/mL) contained about 8.5% apoptotic cells (measured as a sum of early and late apoptotic cells).

### 2.4. Effect of Compounds ***1**–**3*** and Etoposide on Caspase-8 and Caspase-9 Activity

**Figure 3:**
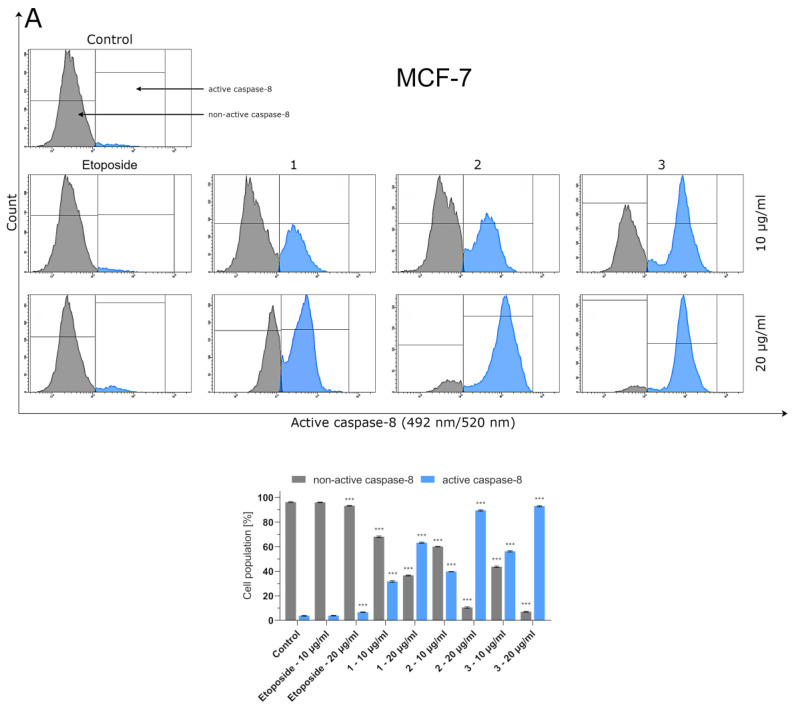

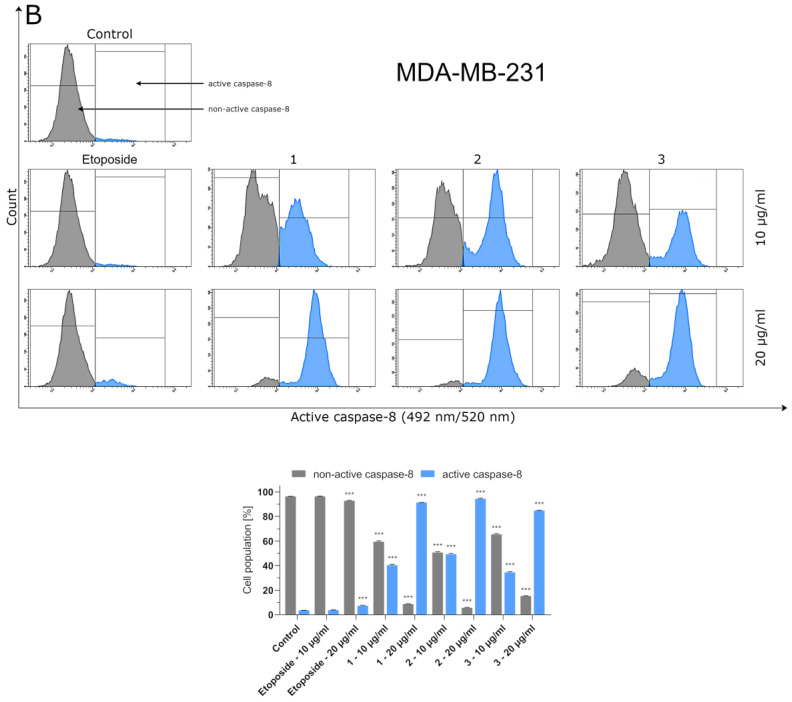
Flow cytometric analyses of MCF-7 (**A**) and MDA-MB-231 (**B**) breast cancer cells treated for 24 h with **1–3** and etoposide for active caspase-8. The compounds were tested at the following concentrations: **1** (10 µg/mL/23.83 µM and 20 µg/mL/47.66 µM), **2** (10 µg/mL/22.75 µM and 20 µg/mL/45.50 µM), **3** (10 µg/mL/23.78 µM and 20 µg/mL/47.56 µM) andetoposide (10 µg/mL/17 µM and 20 µg/mL/34 µM). Mean percentage values from three independent experiments (n = 3) performedin duplicate are presented. *** *p* < 0.001 vs. control group.

**Figure 4:**
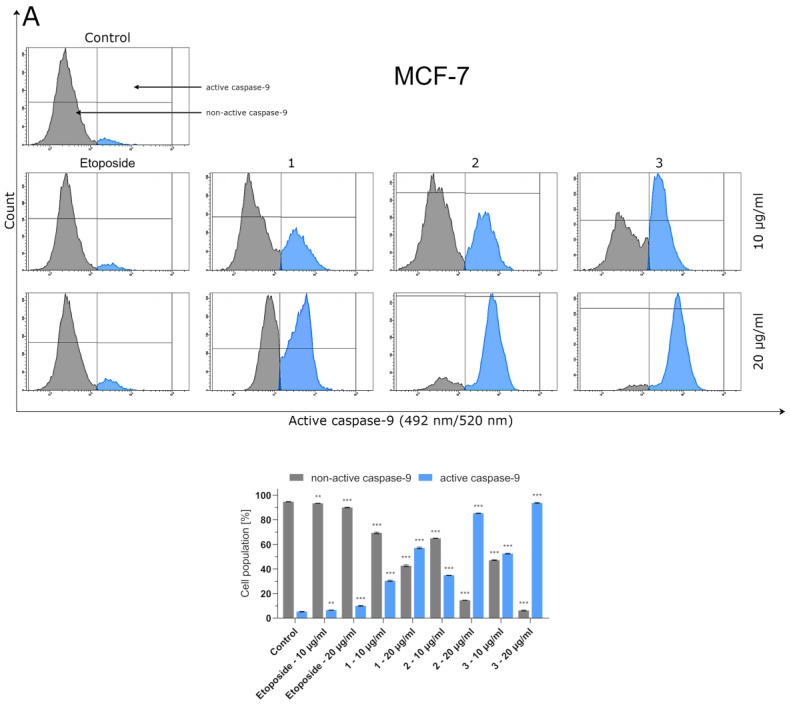

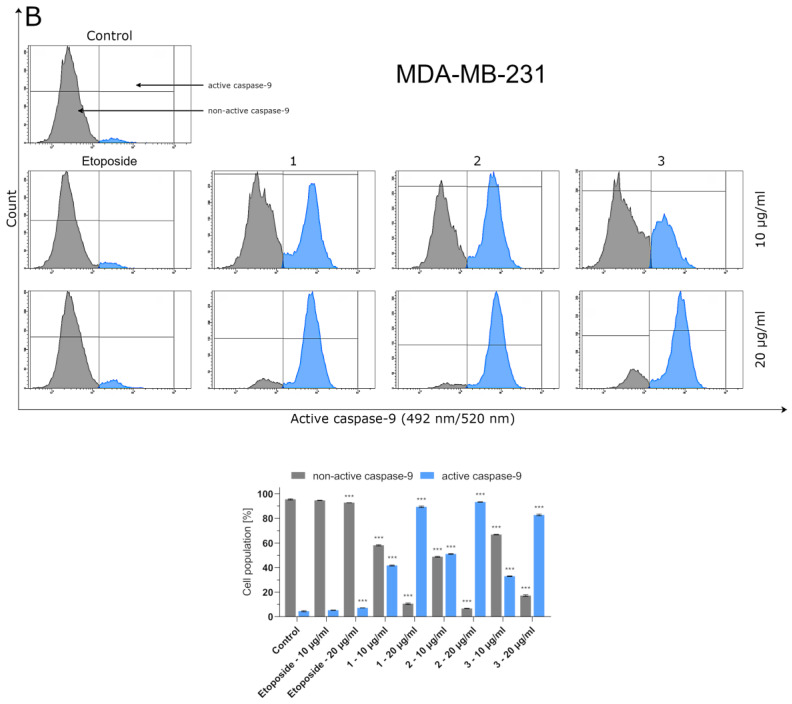
Flow cytometric analyses of MCF-7 (**A**) and MDA-MB-231 (**B**) breast cancer cells treated for 24 h with compounds **1**–**3** and etoposide for active caspase-9. The compounds were tested at the following concentrations: **1** (10 µg/mL/23.83 µM and 20 µg/mL/47.66 µM), **2** (10 µg/mL/22.75 µM and 20 µg/mL/45.50 µM), **3**(10 µg/mL/23.78 µM and 20 µg/mL/47.56 µM) andetoposide (10 µg/mL/17 µM and 20 µg/mL/34 µM). Mean percentage values from three independent experiments (n = 3) performedin duplicate are presented. ** *p* < 0.01 vs. control group, *** *p* < 0.001 vs. control group.

Caspases-8 and -9 are known as initiators of the apoptosis process. However, while activation of caspase-8 is a crucial step in the initiation of the death-receptor-mediated pathway of apoptosis (extrinsic pathway), caspase-9 plays a pivotal role in the intrinsic pathway mediated by mitochondria. Flow cytometric analyses showed that the reference drug (etoposide) statistically significantly increased caspase-8 activation in breast cancer cells only when used at a concentration of 20 µg/mL (Figure 3). However, the number of MCF-7 and MDA-MB-231 cells with activated caspase-8 was still as low as 6.73 and 7.2%, respectively. A significant and dose-dependent increase in caspase-8 activation was observed in breast cancer cells pretreated with compounds **1**–**3**. Among the investigated thiosemicarbazide derivatives, compound **3** had the highest ability to induce caspase-8 activation in MCF-7 cells, while triple-negative MDA-MB-231 breast cancer cells were more sensitive to thiosemicarbazide derivative 2. Similar dependencies were observed when MCF-7 and MDA-MB-231 cells were tested for caspase-9 activity (Figure 4). In order to investigate the ability of compounds **1**–**3** to induce apoptosis-related caspases-8 and -9, MCF-7 and MDA-MB-231 cells were pretreated with pan-caspase inhibitor (Z-VAD-FMK, 100 µM). Caspases-8 and -9 were strongly activated even after previous addition of Z-VAD-FMK (Figures S1 and S2). These observations collectively prove that apoptosis induced by compounds **1**–**3** in breast cancer cells is triggered via both intrinsic and extrinsic pathways.

### 2.5. Thiosemicarbazide Derivative 1 Induces Autophagy in MCF-7 and MDA-MB-231

**Figure 5:**
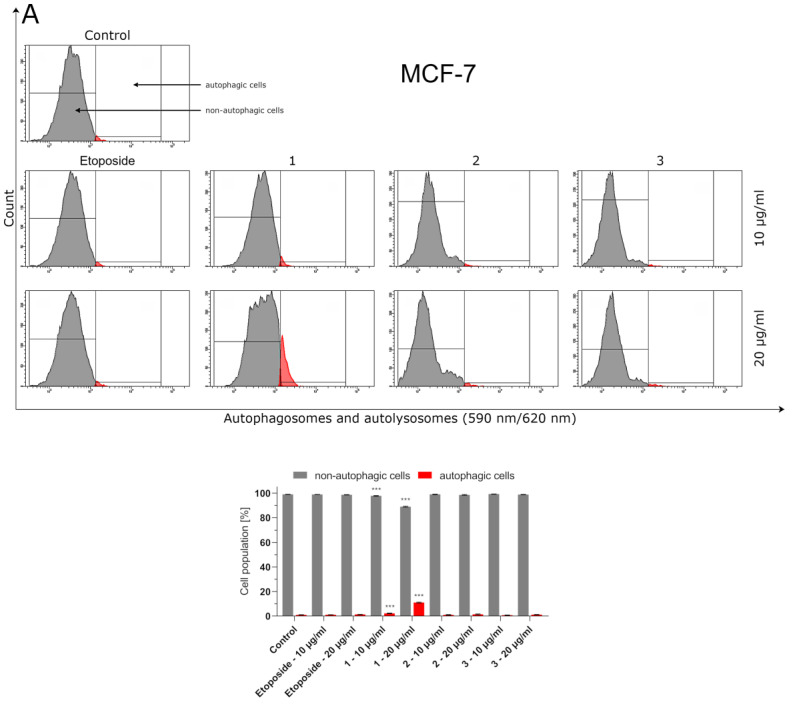

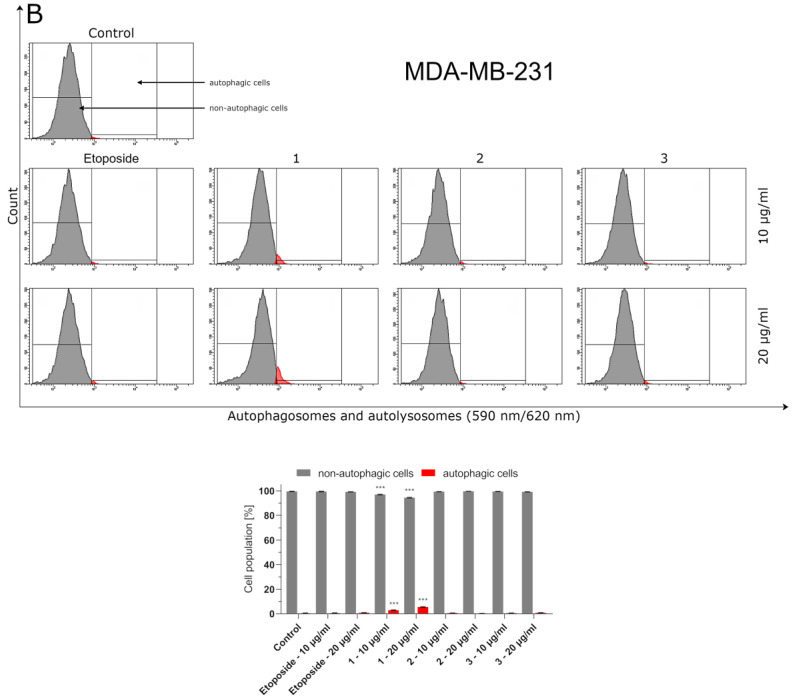
Autophagy induction in MCF-7 (**A**) and MDA-MB-231 (**B**) breast cancer cells measured by flow cytometry using an autophagy probe (red color) compared to negative control cells (gray color) after 24 h of incubation with compounds **1**–**3** and etoposide. The compounds were tested at the following concentrations: **1** (10 µg/mL/23.83 µM and 20 µg/mL/47.66 µM), **2** (10 µg/mL/22.75 µM and 20 µg/mL/45.50 µM), **3** (10 µg/mL/23.78 µM and20 µg/mL/47.56 µM), etoposide (10 µg/mL/17 µM and 20 µg/mL/34 µM). Mean percentage values from three independent experiments (n = 3) performed in duplicate are presented. *** *p* < 0.001 vs. control group.

Besides apoptosis, cancer cells may be successfully eliminated from living organisms through the functionally distinct mechanism of programmed cell death, i.e., through the autophagy process. Cells undergoing autophagy display an increase in the number of autophagosomes and autolysosomes which, subsequently, can be labeled with a fluorescent autophagy probe. Such a method is widely used to monitor the occurrence and progress of autophagy. Among the investigated thiosemicarbazide derivatives, only compound **1** induced the autophagy process in MCF-7 and MDA-MB-231 breast cancer cells in a statistically significant manner. In the population of MCF-7 cells pretreated with 10 and 20 µg/mL of compound **1**, 2.5 ± 0.2% and 11.5 ± 1.2% of cells were observed to beundergoing autophagy, respectively (Figure 5). In the case of MDA-MB-231 cells, these values ranged from 3.0 ± 0.2% to 5.5 ± 0.9%. Regarding both untreated and etoposide-treated breast cancer cells, as low as about 1% of the cell population formed autophagosomes and/or autolysosomes.

### 2.6. Thiosemicarbazide Derivatives 1–3 Trigger Cell Cycle Arrest in MCF-7 and MDA-MB-231 Breast Cancer Cells

**Figure 6:**
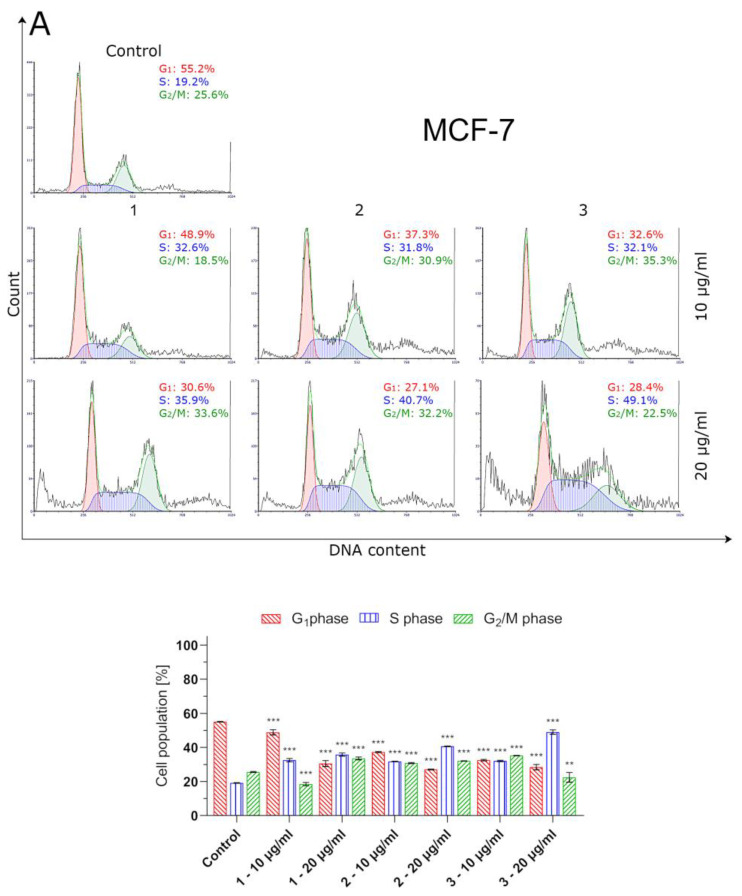

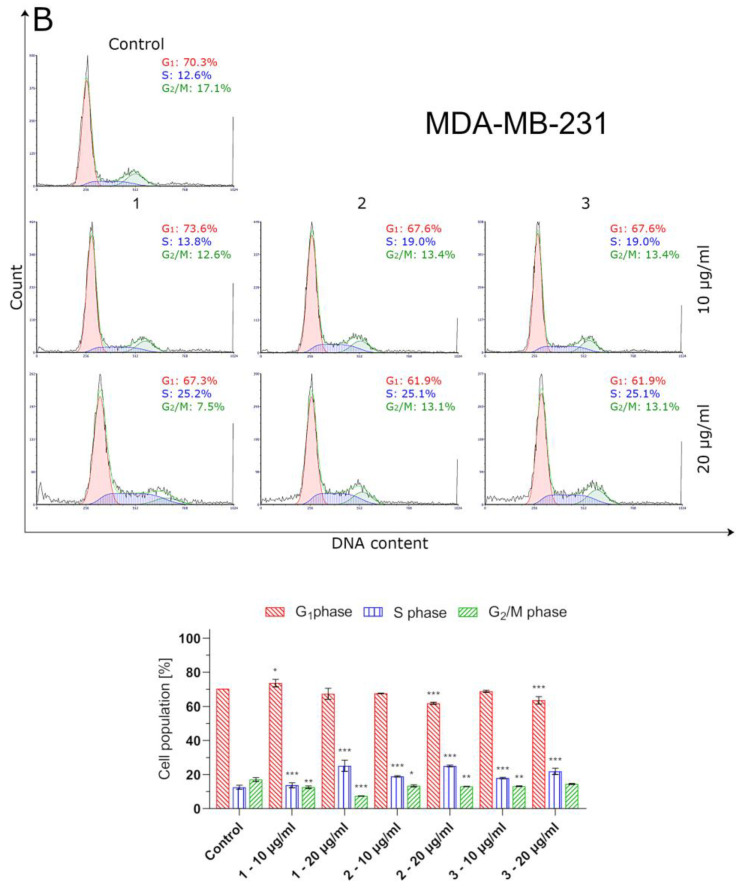
Cell cycle analyses of MCF-7 (**A**) and MDA-MB-231 (**B**) breast cancer cells incubated for 24 h with compounds **1**–**3**. The compounds were tested at the following concentrations: **1** (10 µg/mL/23.83 µM and 20 µg/mL/47.66 µM), **2** (10 µg/mL/22.75 µM and 20 µg/mL/45.50 µM), **3**(10 µg/mL/23.78 µM and 20 µg/mL/47.56 µM) andetoposide(10 µg/mL/17 µM and 20 µg/mL/34 µM). Mean percentage values from three independent experiments (n = 3) performed in duplicate are presented. * *p* < 0.05 vs. control group, ** *p* < 0.01 vs. control group, *** *p* < 0.001 vs. control group.

The investigated compounds impaired cell cycle progression to a much greater extent in MCF-7 cells. However, they caused S-phase cell cycle arrest in a dose-dependent manner, both in MCF-7 and MDA-MB-231 cells. After 24 h of culture with higher concentrations of 1–3 (20 µg/mL), the percentage of MCF-7 cells in the S-phase increased from 19.2% (control cells) to 35.9% (1), 40.7% (2) and 49.1% (3). The number of S-phase arrested MCF-7 cells was nearly twice as high as MDA-MB-231 cells pretreated with thiosemicarbazide derivatives 1–3 (20 µg/mL). Additionally, an increased proportion of G2/M phase cells was observed when MCF-7 cells were incubated with 20 µg/mL of 1 (33.6%) and 2 (32.2%) or 10 µg/mL of 3 (35.3%). Under the same conditions of cell culture, the number of control (untreated) MCF-7 cells in theG2/M phase was 25.6% (Figure 6).

### 2.7. Effect of Compounds ***1**–**3*** on the Activity of ATP-Binding Cassette (ABC) Transporters

**Figure 7:**
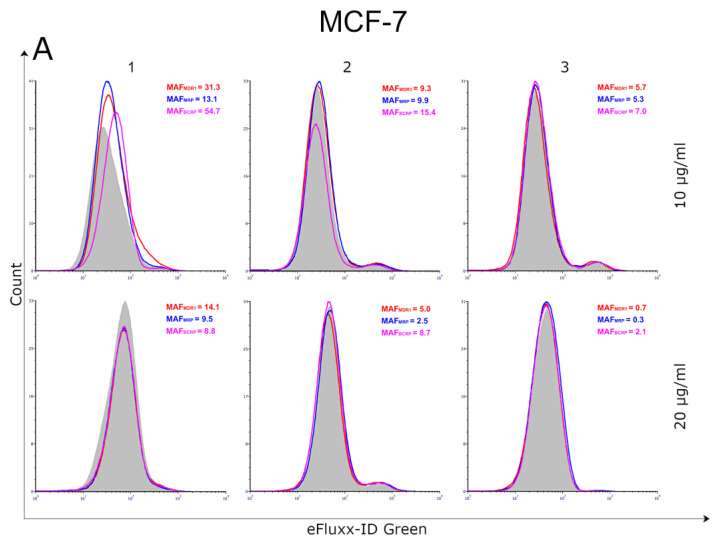

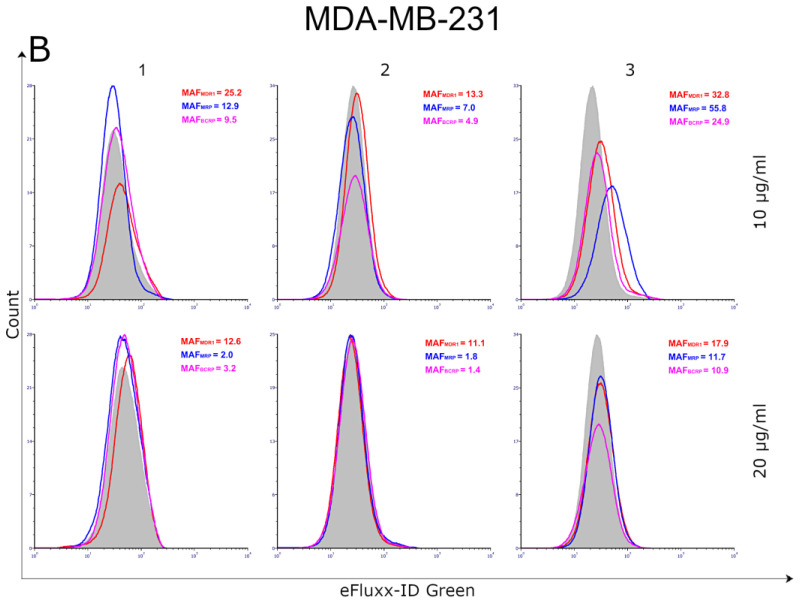
Flow cytometry analyses of ABC transporters in MCF-7 (**A**) and MDA-MB231 (**B**) cell pretreated with compounds **1–3**. The compounds were tested at the following concentrations: 1 (10 µg/mL/23.83 µM and 20 µg/mL/47.66 µM), 2 (10 µg/mL/22.75 µM and20 µg/mL/45.50 µM), 3 (10 µg/mL/23.78 µM and 20 µg/mL/47.56 µM) and etoposide (10 µg/mL/17 µM and 20 µg/mL/34 µM).Cells were incubated with Efflux Green Detection Reagent, with and without specific inhibitors (Verapamil, MK-571 and Novobiocin). Gray histograms show the fluorescence of the sample without an inhibitor, while tinted histograms show the fluorescence of cells treated with appropriate inhibitors (MDR1—red histogram, MRP—blue histogram and BCRP—pink histogram). The difference in fluorescence is indicative of the protein activity. The numbers in the upper right corners are MAF scores (multidrug resistance activity factors) that quantitatively characterize the multidrug resistance.

ABC transporters play a pivotal role in the development of resistance to anticancer treatment. Therefore, the effect of thiosemicarbazide derivatives 1–3 on the activity of three major ATP-binding cassette (ABC) transporter proteins, i.e., MDR1 (also known as *p*-glycoprotein), MRP 1/2 (multidrug resistance-associated protein) and BCRP (breast cancer resistance protein), was evaluated. The quantitative analyses of ABC transporter activity were expressed using multidrug-resistance activity factor (MAF) values. Studies comparing MAF values with clinical responses to chemotherapy have suggested that agents with an MAF of <20 can be regarded as multidrug-resistance negative, while MAF values of >25 are indicative of multidrug-resistance positive specimens. The current studies revealed that the investigated compounds inhibited the activity of MDR1, MRP1/2 and BCRP transporters in a dose-dependent manner. Compounds **2** and **3**, stronger than compound **1**, decreased the activity of the examined protein transporters in MCF-7 cells. Even at the lower concentrations tested (i.e., 10 µg/mL), the former thiosemicarbazide derivatives impaired the ABC transporter activity, with MAF values much lower than 20 (for compound **2**: MAF_MDR1_ = 9.1, MAF_MRP_ = 9.9 and MAF_BCRP_ = 15.4 and for compound **3**: MAF_MDR1_ = 5.7, MAF_MRP_ = 5.3 and MAF_BCRP_ = 7.0). In the case of MDA-MB-231 cells, the highestinhibitory potency against MDR1, MRP1/2 and BCRP transporter proteins was exhibited by compound **2** (at 10 µg/mL: MAF_MDR1_ = 13.3, MAF_MRP_ = 7.0 and MAF_BCRP_ = 4.9; at 20 µg/mL: MAF_MDR1_ = 11.1, MAF_MRP_ = 1.8 and MAF_BCRP_ = 1.4). However, pretreatment of MDA-MB-231 cells with thiosemicarbazide derivatives 1 and 3 at a concentration of 20 µg/mL also resulted in the significantly diminished activity of the ABC transporter proteins, with MAF values less than 20 (Figure 7).

### 2.8. Screening of ADME-Tox Properties of Thiosemicarbazide Derivatives 1–3

**Figure 8:**
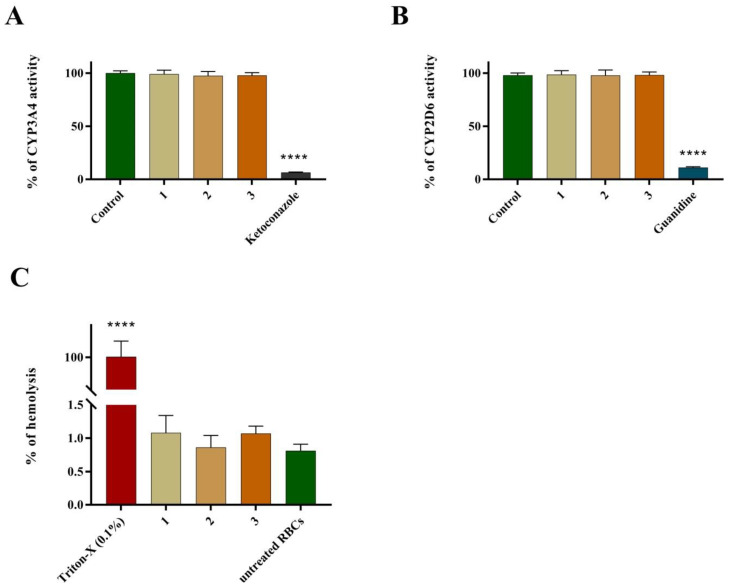
ADME-Tox properties of compounds **1–3** tested at a concentration of 10 µg/mL: (**A**) effect on CYP3A4 activity, (**B**) effect on CYP2D6 activity and (**C**) hemolytic activity determined inhuman red blood cells. Data are presented as a percentage of control activity (%). Ketoconazole and guanidine (positive controls) were tested at a concentration of 10 µM. **** *p* < 0.0001 (one-way ANOVA).

CYP3A4 and CYP2D6 enzymatic activities were evaluated using Vivid™ CYP3A4 and CYP2D6 screening kits with ketoconazole and guanidine as positive controls, respectively. None of the tested compounds influenced the activity of CYP3A4 and CYP2D6 enzymes at a concentration of 10 µg/mL in a statistically significant manner. At the same time, ketoconazole (10 µM) decreased the activity of CYP3A4 to 6.49 ± 0.38% (vs. the negative control), while guanidine significantly impaired the activity of CYP2D6 (11.08 ± 0.81% vs. negative control) (Figure 8A,B). The hemolytic effect of 1–3 varied from 0.86 ± 0.18% (compound **2**) to 1.08 ± 0.26% (compound **1**); however, there were no statistically significant differences between those results and hemolysis observed in untreated red blood cells (0.81 ± 0.11%) (Figure 8C).

## 3. Discussion

Small molecule inhibitors still constitute an integral part of chemotherapy regimens. Among such molecules, special attention is given to dual- or multi-targeting agents, whose single molecules are able to act on multiple molecular targets [15,16]. One of their main advantages, when compared to anticancer agents that have a large molecular weight, is that they can pass through the cell membranes and interact with the intracellular targets. The use of dual- or multi-targeting anticancer agents allows to overcome the problems of limited efficacy or chemoresistance, observed when single-targeting agents are applied. Thiosemicarbazide derivatives 1–3 (Figure 1), recently designed and synthesized in our laboratory, constitute examples of dual-targeting cytotoxic agents acting on human DNA topoisomerase IIα and indoleamine-2,3-dioxygenase. Previously, it was shown that these compounds significantly reduced the viability of both ER/PR-positive MCF-7 cells and triple-negative MDA-MB-231 cells [4]. The anti-BC activities of 1–3 were higher than that of etoposide, which is a marketed anticancer drug targeting human topoisomerase IIα. Importantly, current studies revealed that BC cells were more sensitive to 1–3 than normal breast epithelial cells (MCF-10A), with SI values of up to 5.6. Another beneficial effect of the investigated compounds, apart from their cytotoxic activity and selectivity towards BC cells, is associated with the reduction in the ABC transporter activity. This is crucial, because despite the clinical efficacy of anticancer agents acting as topoisomerase II poisons, their use in cancer treatment has significant limitations associated with the development of drug resistance [17]. Activation or overexpression of the ATP-binding cassette proteins, associated with the pumping of drugs from cancer cells, result in a decreasedresponse to chemotherapy [18,19,20]. The main role in the development of chemoresistance in BC cells is played by MDR1 (*p*-glycoprotein) and subsequently by MRP (multidrug resistance-associated proteins) and BCRP (breast cancer resistance protein). Their improved activity correlates with cancer aggressiveness, progression of the disease and a poor prognosis [21]. The dose-dependent inhibition of the investigated ABC transporters (MDR1, MRP1/2 and BCRP) in MCF-7 and MDA-MB-231 cells by compounds **1–3** impliesalower risk of drug resistance. Interestingly, the compounds withthe lowest MAF values (i.e., compound **3** against MCF-7 cells and compound **2** against MDA-MB-231 cells) were also characterized by lower IC_50_ values established in MTT assays.

Bearingin mind that topoisomerase inhibitors belong to the most effective inducers of apoptosis, the possible pro-apoptotic effect of 1–3 in BC cells was investigated. Using MCF-7 and MDA-MB-231 cells, it was demonstrated that the investigated thiosemicabazide derivatives promoted apoptosis in a manner correlated to their IC_50_ values. In the case of topoisomerase II poisons, such a phenomenon can be explained as a result of intracellular accumulation of covalent topoII-DNA complexes. So-called “cleavable complexes” act as cellular toxins that block DNA replication and stimulate apoptotic pathways [22]. The data from the current study show that the pro-apoptotic effect produced by compounds **1–3** is mediated via activation of caspases-8 and -9, and thus involves both extrinsic and intrinsic apoptotic pathways in MCF-7 and MDA-MB-231 cells. Additionally, the autophagy process was observed in both types of investigated BC cells after incubation with compound **1**. Autophagy itself is considered to be a double-edged sword. Depending on the type of cancer, its stage, genetic factors and the treatment applied, autophagy can suppress tumorigenesis or promote cancer cell survival and proliferation [23,24]. In our studies, the rate of autophagy in MCF-7 cells was nearly twice as high as in MDA-MB-231 cells. Importantly, some research suggests the existence of the antagonism between autophagy and apoptosis [25]. This may explain why compound **1**, which turned out to be an autophagy inducer in MCF-7 and MDA-MB-231 cells, also had the weakest promoting effect on apoptosis out of the three thiosemicarbazide derivatives tested. However, the possible involvement of autophagy in compound **1**-induced BC cell death requires further investigation and cannot be claimed at this stage of the study. Apart from the contribution of apoptosis and ABC transporter inhibition to the anticancer effect of 1–3, our studies also showed that these dual-targeting agents significantly inhibited the proliferation of BC cells by blocking cell cycle in the S-phase in a dose-dependent manner. The observed impairment in cell cycle progression was significantly stronger in MCF-7 cells. Thus, it seems clear that the disruption of so many intracellular processes results in efficient suppression of BC cells. 

Administration of cancer therapy includes several routes, e.g., intravenous, oral, intraperitoneal, intrathecal, subcutaneous, intradermal, etc. However, chemotherapy is most commonly given intravenously. Although very rarely, anticancer agents may cause direct (toxic) or delayed (immunological) hemolytic effects. Therefore, it is of great importance to test the possible hemolytic activity of drug candidates at the earliest possible stage of their development. Drug-induced hemolysis can be mediated either by immune- or non-immune mechanisms. The latter mechanismis more likely in patients with glucose-6-phosphate dehydrogenase (G6PD)deficiency, since this enzyme protects red blood cells from oxidative stress-related damages. The investigated thiosemicarbazides 1–3 did not result indirect hemolysis mediated by non-immune mechanisms after incubation with red blood cells in vitro. As a limitation of this study, it should be pointed out that the possible immune-related hemolytic effects of 1–3 could only be observed in in vivo conditions. Therefore, additional experiments are required to completely exclude the hemolytic potential of the tested drug candidates.

During further testing of ADME-Tox properties of 1–3, their effects on specific cytochrome P450 (CYP) enzymes (i.e., CYP3A4 and CYP2D6) were evaluated. Preliminary safety assessments of drug candidates in terms of their possible risk of CYP induction/inhibition remains pivotal in drug discovery and development. CYP enzymes are responsible for biotransformation of drugs and other xenobiotics. Their inhibition or overactivation may lead to drug–drug interactions, causing toxic effects and/or therapeutic failure. Among the CYP enzymes, CYP3A4 and CYP2D6 isoforms are the most essential. Taken together, they are responsible for the metabolism of approximately 80% of the available drugs (CYP3A4 metabolizes approx. 50% of drugs, while CYP2D6 metabolizes approx. 30%) [26]. After incubation of CYP3A4 and CYP2D6 in the presence of compounds **1–3** (10µg/mL), no statistically significant changes in the activity of the tested enzymes were observed. Therefore, it can be concluded that the possible use of thiosemicarbazide derivatives 1–3 will be associated with a low risk of drug–drug interactions.

## 4. Materials and Methods

### 4.1. Cell Culturing

Human breast cancer cells, i.e., MCF-7 (estrogen-dependent breast cancer cells) and MDA-MB-231 (estrogen-independent breast cancer cells), as well as normal human breast epithelial cells (MCF-10A) were purchased from the American Type Culture Collection (ATCC, Manassas, VA, USA). MCF-7 and MDA-MB-231 cells were cultured in DMEM (Sigma Aldrich, St. Louis, MO, USA) supplemented with 10% fetal bovine serum (FBS), penicillin (100 U/mL) and streptomycin (100 μg/mL). MCF-10A cells were cultured in Mammary Epithelial Cell Growth Medium supplemented with BPE, hEGF, insulin, hydrocortisone, GA-1000 (Lonza, Basel, Switzerland), 10% fetal bovine serum (FBS), penicillin (100 U/mL) and streptomycin (100 μg/mL). The cells were grown in T25 Cell Culture EasyFlask (Nunc, Roskilde, Denmark) at 37 °C in a humidified atmosphere of 5% CO_2_.

### 4.2. Effect of Compounds ***1**–**3*** on the Viability of MCF-10A Cells

MTT assays were performed to evaluate the cytotoxic activity of the investigated compounds (**1**–**3**) against MCF-10A cells. Asuspension of cells (1 × 10^5^ cells/mL) was distributed onto 96-well plates at a volume of 100 μL/well. After attachment, the cells were treated with increased concentrations of the tested compounds in medium containing 2% FBS, and incubated for 24 h. Then, 15 μL of MTT working solution (5 mg/mL in PBS) was added to each well and the plates were incubated for 3 h. Subsequently, 100 μL of 10% SDS solution was added to each well in order to dissolve the formazan formed. After overnight incubation at 37 °C, the absorbance of the obtained solution was measured at λ= 570 nm using a microplate reader (Epoch, BioTek Instruments, Inc., Winooski, VT, USA). At least two independent experiments were performed in triplicate. IC_50_ values of the tested compounds were calculated using the IC_50_ online calculator [27].

### 4.3. Flow Cytometry Assessment of Annexin V and Propidium Iodide Binding

Flow cytometry analyses for apoptosis induction by the compounds **1**–**3** and etoposide wereperformedusing an FITC Annexin V Apoptosis Detection Kit II (BD Pharmingen, San Diego, CA, USA). The rationale of the assay is based on the externalization of phosphatidylserine on the surface of apoptotic cells. In early and late apoptotic cells, the phosphatidylserine translocates into the outer layer of the membrane, which allows annexin V-FITC to attach. Propidium iodide (PI) is, in turn, a fluorescent dye that penetrates into the late apoptotic and necrotic cells with impaired cell membrane integrity. Therefore, using annexin V and PI, it is possible to distinguish four groups of cells: living cells, early apoptotic cells, late apoptotic cells and necrotic cells. MCF-7 and MDA-MB-231 cells were incubated with 10 and 20 µg/mL of compounds **1**–**3** and etoposide (reference drug) for 24 h. After incubation, the cells were dyed with FITC-labeled annexin V and PI. The analysis was performed on a BD FACS Canto II flow cytometer using FACSDiva software (version 6.1.3, BD Biosciences Systems, San Diego, CA, USA). The equipment was calibrated with BD Cytometer Setup and Tracking Beads (BDBiosciences, San Diego, CA, USA).

### 4.4. Effect of Compounds ***1**–**3*** and Etoposide on the Authophagy Process in MCF-7 and MDA-MB-231 Cells

In order to evaluate the number of autophagosomes and autolysosomes in breast cancer cells incubated with the investigated compounds, anAutophagy Assay (Red kit) was performed (ImmunoChemistry Technologies, Bloomington, MN, USA). In short, the unfixed cells were washed and resuspended in PBS with the added autophagy probe, Red solution. Next, the cells were incubated for 30 min at 37 °C in the dark, washed, resuspended in cellular assay buffer and the provided fixative was added at a volume/volume ratio of 1:5. The samples were measured immediately after preparation by flow cytometry using the BD FACSCanto II system (BD Biosciences Systems, San Diego, CA, USA). The percentage of cells with autophagy was calculated using FACSDiva software (version 6.1.3, BD Biosciences Systems, San Diego, CA, USA).

### 4.5. Cell Cycle Analysis

Cell cycle analyses of MCF-7 and MDA-MB-231 cells treated with compounds **1**–**3** and etoposide for 24 h were performed using a FACSCanto II flow cytometer (BD Bioscences Systems, San Diego, CA, USA). After incubation, breast cancer cells were trypsinized and fixed with cold ethanol (70%) and stored overnight at −20 °C. After removing ethanol, the cells were washed three times with PBS, treated with 50 μg/mL of DNase-free RNase A Solution (Promega, Madison, WI, USA) and stained with 100 μg/mL of PI. The results of measurements were analyzed using FCS Express 7 software (De Novo Software, Pasadena, CA, USA).

### 4.6. Caspase Enzymatic Activity Assay

Caspase-8 and caspase-9 activitiesweremeasured using the FLICA Caspase-8 Assay Kit and FLICA Caspase-9 Assay Kit (ImmunoChemistry Technologies, Bloomington, MN, USA), respectively, according to the manufacturer’s protocol. In brief, MCF-7 and MDA-MB-231 breast cancer cells (1 × 10^6^) were washed with cold PBS twice and re-suspended in buffer. Then, 5 μL of diluted FLICA reagent and 2 μL of Hoechst 33,342 were added to 93 μL of cell suspension, mixed by pipetting and incubated for 60 min at 37 °C. After incubation, the cells were washed twice with 400 μL of apoptosis wash buffer and centrifuged at 300× *g*. After the last wash, the cells were resuspended in 100 μL of apoptosis wash buffer supplemented with 10 μg/mL of PI. Analyses were performed using aBD FACSCanto II flow cytometer, and the results were analyzed with FACSDiva software (version 6.1.3, BD BiosciencesSystems, San Diego, CA, USA). To identify the involvement of caspases in apoptosis induced by compounds **1**–**3**, the investigated breast cancer cells were pretreated with the pan-caspase inhibitor, Z-VAD-FMK (100 µM) (Sigma Aldrich, St. Louis, MO, USA), as described in [28]. The activation of caspases-8 and -9 was detected as described above.

### 4.7. Multidrug Resistance (MDR) Transporter Activity Evaluation

An MDR Assay kit (Abcam, Cambridge, UK) was used following the manufacturer’s instructions. MCF-7 and MDA-MB-231 cells were incubated with compounds **1**–**3** (10 μg/mL and 20 μg/mL). After 24 h of incubation, the test cells were washed twice with 5 mL of PBS by centrifugation at 1200 rpm for 10 min. The supernatants were discarded and the cells were counted using a Scepter 3.0 handheld automated cell counter (Milipore, Burlington, MA, USA). Then, each of the tested samples was divided into three parts and analyzed. Tomeasure the activity of MDR1, MRP1 and BCRP (tubes 1–12), 5 μL of verapamil (MDR1 inhibitor) was added into tubes 1–3, 5 μL of MK-571 (MRP1 inhibitor) was added into tubes 4–6, 5 μL of noviobiocin (BCRP inhibitor) was added into tubes 7–9 and 125 μL DMEM with 1 μL of DMSO was added into tubes 10–12. Then, the samples were incubated at 37 °C for five minutes. After this time, 125 μL of Efflux Green Detection Reagent was added into tubes 1–12 and the samples were incubated for 30 min at 37 °C. After 25 min of incubation, 5 μL of PI was added to each tube. Thereafter, the samples were centrifuged for 10 min at 1200 rpm. The supernatants were discarded and the cells were resuspended in 500 μL of PBS and run on the flow cytometer immediately. Measurements were conducted on a BD FACSCanto II flow cytometer using FACSDiva software (version 6.1.3, BD Biosciences, San Diego, CA, USA). The multidrug resistance activity factor (MAF) was calculated from the difference between the mean fluorescence intensity (MFI) of cells with and without the highly selective inhibitors. The calculations followed these formulas:

MAF_MDR1_= 100 × (F_MDR1_ − F_0_)/F_MDR1_

MAF_MRP_= 100 × (F_MR_P − F_0_)/F_MRP_

MAF_BCRP_= 100 × (F_BCRP_ − F_0_)/F_BCRP_

F_MDR_—MFI with MDR1 inhibitor (verapamil)
F_MRP_—MFI with MRP inhibitor (MK-571)
F_BCRP_—MFI with BCRP inhibitor (novobiocin)
F_0_—MFI without inhibitor

The theoretical range of the MAF values is between 0 and 100. Studies comparing MAF values with the clinical response to a chemotherapeutic treatment suggest that a specimen with an MAF value of <20 can be regarded as multidrug resistance negative, while MAF values of >25 are indicative of multidrug resistance positive specimens.

### 4.8. Hemolytic Activity of Compounds ***1**–**3***

Human red blood cell (RBC) concentrate was obtained from the Regional Blood Donation and Transfusion Centre (Lublin, Poland). The RBC concentrate (5 mL) was washed three times with sterile PBS at 37 °C and centrifuged at 500 g for 3 min. The obtained pellet was resuspended using warm, sterile PBS in order to obtain a 2% RBC suspension. Next, 1 mL of the RBC suspension was mixed with 1 mL of a solution of the investigated compounds. The final concentration of the tested compounds was 10 µg/mL. The mixtures were incubated at 37 °C for 30 min and centrifuged at 1500× *g* for 10 min. Finally, avolume of 100 µL of supernatant from each sample was transferred into a 96-well plate and the amount of free hemoglobin was measured spectrophotometrically at 405 nm. PBS alone (negative control) and 0.1% Triton-X (positive control) were examined using similar conditions. The experiments were run in triplicate.

### 4.9. Effect of Compounds ***1**–**3*** on CYP3A4/CYP2D6 Enzymatic Activity

CYP3A4 and CYP2D6 enzymatic activities were evaluated using a Vivid™ CYP3A4 Green Screening Kit and a Vivid™ CYP2D6 Cyan Screening Kit (both from Thermo Fisher Scientific, Waltham, MA, USA), respectively, according to the manufacturer’s protocol. According to these assays, the examined compounds are analyzed by their ability to inhibit the production of a fluorescent signal in the reaction between recombinant CYP3A4 or CYP2D6 isozymes and specific Vivid substrates. The experiments were performed in the endpoint mode of the assay, in which solutions of examined compounds were first combined with the Master Pre-mix. After a short incubation, the background fluorescence of the Master Pre-mix and the examined compounds was measured. Next, a mix of the respective Vivid substrate and NADP^+^ was added in order to initiate the enzymatic reaction. After 20 min of incubation, the reaction was terminated using a stop solution and the fluorescence was measured using the recommended (by the manufacturer) excitation and emission wavelengths. Ketoconazole and quinidine (both at 10 µM) were used as positive controls (i.e., inhibitors of CYP3A4 and CYP2D6, respectively). A reaction mixture without tested compounds was used as a negative control and its fluorescence was designated as 100%. The experiments were run in triplicate and the results were presented as means ± SD.

## 5. Conclusions

This paper provides an insight into the anticancer effects of three topoIIα/IDO 1 inhibitors against breast cancer cell lines. The investigated compounds **1**–**3** selectively inhibited MCF-7 and MDA-MB-231 cells, with selectivity indexes ranging from 2.57 to 5.6 (vs. normal breast epithelial cells). A cytometric analysis revealed that compounds **1**–**3** promoted apoptosis in MCF-7/MDA-MB-231 breast cancer cells via caspase 8/9-related pathways and induced S-phase cell cycle arrest. A slight activation of autophagy by compound **1** was also observed. Bearingin mind that multidrug resistance is often associated with cancer therapy failure, it seems important that topoIIα/IDO 1 inhibitors 1–3 dose-dependently inhibited the activity of ATP-binding cassette transporters in MCF-7/MDA-MB-231 breast cancer cells. Compounds **1**–**3** were also characterized by beneficial ADME-Tox properties, since they did not affect CYP3A4 and CYP2D6 activities and hemolysis.

## 6. Patents

Compounds **1–3** are currently patent pending (WIPO ST 10/C PL439884; WIPO ST 10/C PL439883; WIPO ST 10/C PL439882).

## Figures and Tables

**Table 1:** Effect of compounds **1**–**3**, expressed as IC_50_ values, on the viability of normal MCF-10A cells.

		MCF-10A	MCF-7 *	MDA-MB-231 *	SI_MCF-7_	SI_MDA-MB-231_
**1**	(µg/mL)	25.31 ± 2.27	9.82 ± 0.47	9.21 ± 0.21	2.57	2.75
	(µM)	60.32 ± 5.41	23.40 ± 1.12	21.95 ± 0.5		
**2**	(µg/mL)	42.74 ± 3.16	9.18 ± 0.26	7.64 ± 0.41	4.65	5.60
	(µM)	97.24 ± 7.19	20.88 ± 0.59	17.38 ± 0.93		
**3**	(µg/mL)	40.45 ± 4.59	7.67 ± 0.57	10.47 ± 0.73	5.30	3.86
	(µM)	96.21 ± 10.92	18.24 ± 1.36	24.90 ± 1.74		

SI_MCF-7_—selectivity index calculated for MCF-7 cell line (SI_MCF-7_ = IC_50 MCF-10A_/IC_50 MCF-7_); SI_MDA-MB-231_—selectivity index calculated for MDA-MB-231 cell line (SI_MDA-MB-231_ = IC_50 MCF-10A_/IC_50 MDA-MB-231_); * IC50 values for MCF-7 and MDA-MB-231 cells were taken from our previous paper [4].

## Data Availability

The data presented in this study are available on request from the corresponding author.

## Supplementary Materials

The following supporting information can be downloaded at: https://www.mdpi.com/article/10.3390/ijms24065812/s1.
