# Supplementary material for: Thiosemicarbazide Derivatives Targeting Human TopoIIα and IDO-1 as Small-Molecule Drug Candidates for Breast Cancer Treatment
Source: Int J Mol Sci. 2023 Mar 18;24(6):5812. doi: 10.3390/ijms24065812 (PMC10051798; doi:10.3390/ijms24065812)
Supplement: Supplementary file 1 [file ijms-24-05812-s001.zip › ijms-2165276-supplementary.pdf]

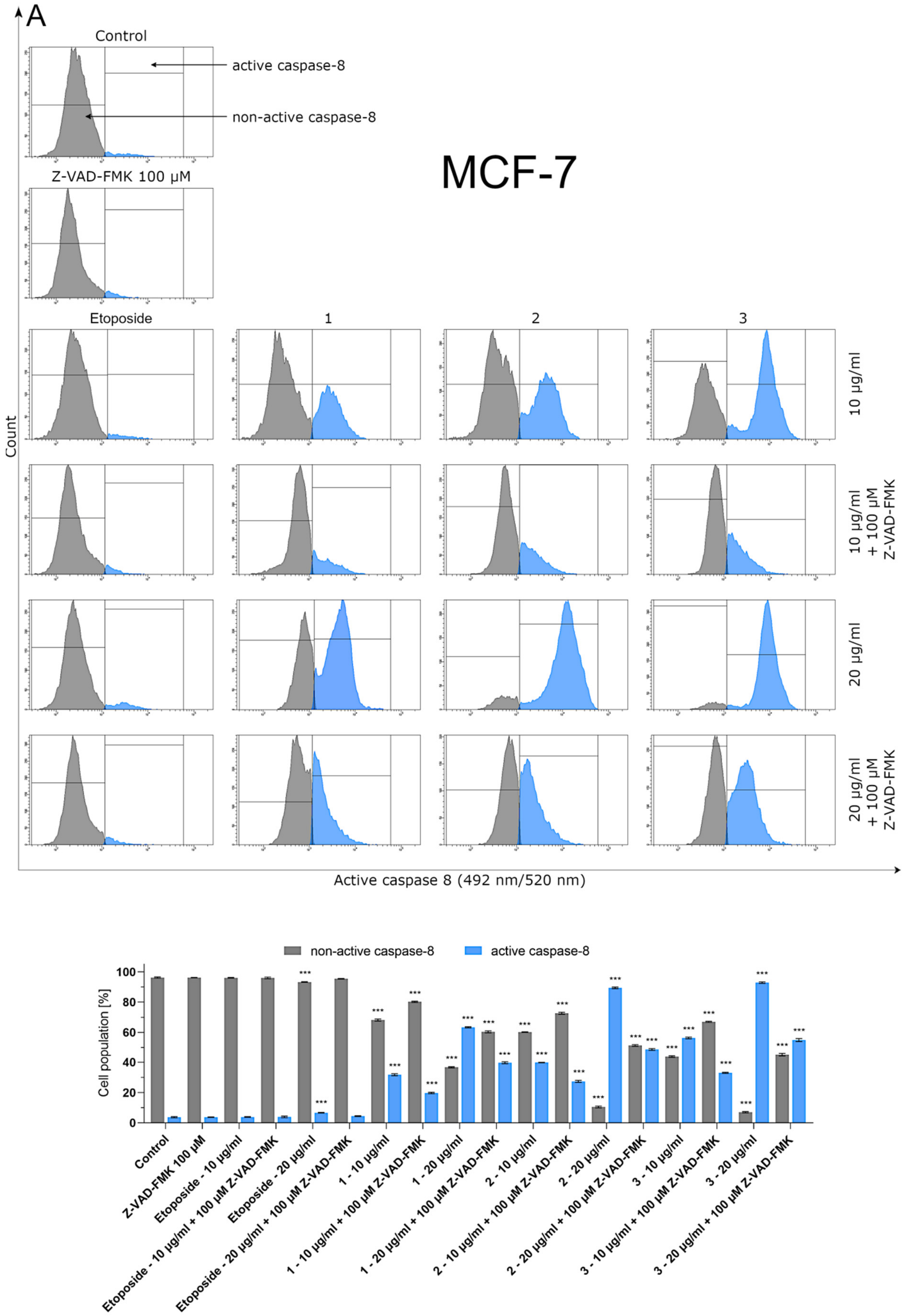
A
MCF-7
Control
active caspase-8
non-active caspase-8
Z-VAD-FMK 100 µM
Etoposide
1
2
3
10 µg/ml
10 µg/ml + 100 µM Z-VAD-FMK
20 µg/ml
20 µg/ml + 100 µM Z-VAD-FMK
Count
Active caspase 8 (492 nm/520 nm)
non-active caspase-8
active caspase-8
Cell population [%]
Control
Z-VAD-FMK 100 µM
Etoposide - 10 µg/ml
Etoposide - 10 µg/ml + 100 µM Z-VAD-FMK
Etoposide - 20 µg/ml
Etoposide - 20 µg/ml + 100 µM Z-VAD-FMK
1 - 10 µg/ml
1 - 10 µg/ml + 100 µM Z-VAD-FMK
1 - 20 µg/ml
1 - 20 µg/ml + 100 µM Z-VAD-FMK
2 - 10 µg/ml
2 - 10 µg/ml + 100 µM Z-VAD-FMK
2 - 20 µg/ml
2 - 20 µg/ml + 100 µM Z-VAD-FMK
3 - 10 µg/ml
3 - 10 µg/ml + 100 µM Z-VAD-FMK
3 - 20 µg/ml
3 - 20 µg/ml + 100 µM Z-VAD-FMK

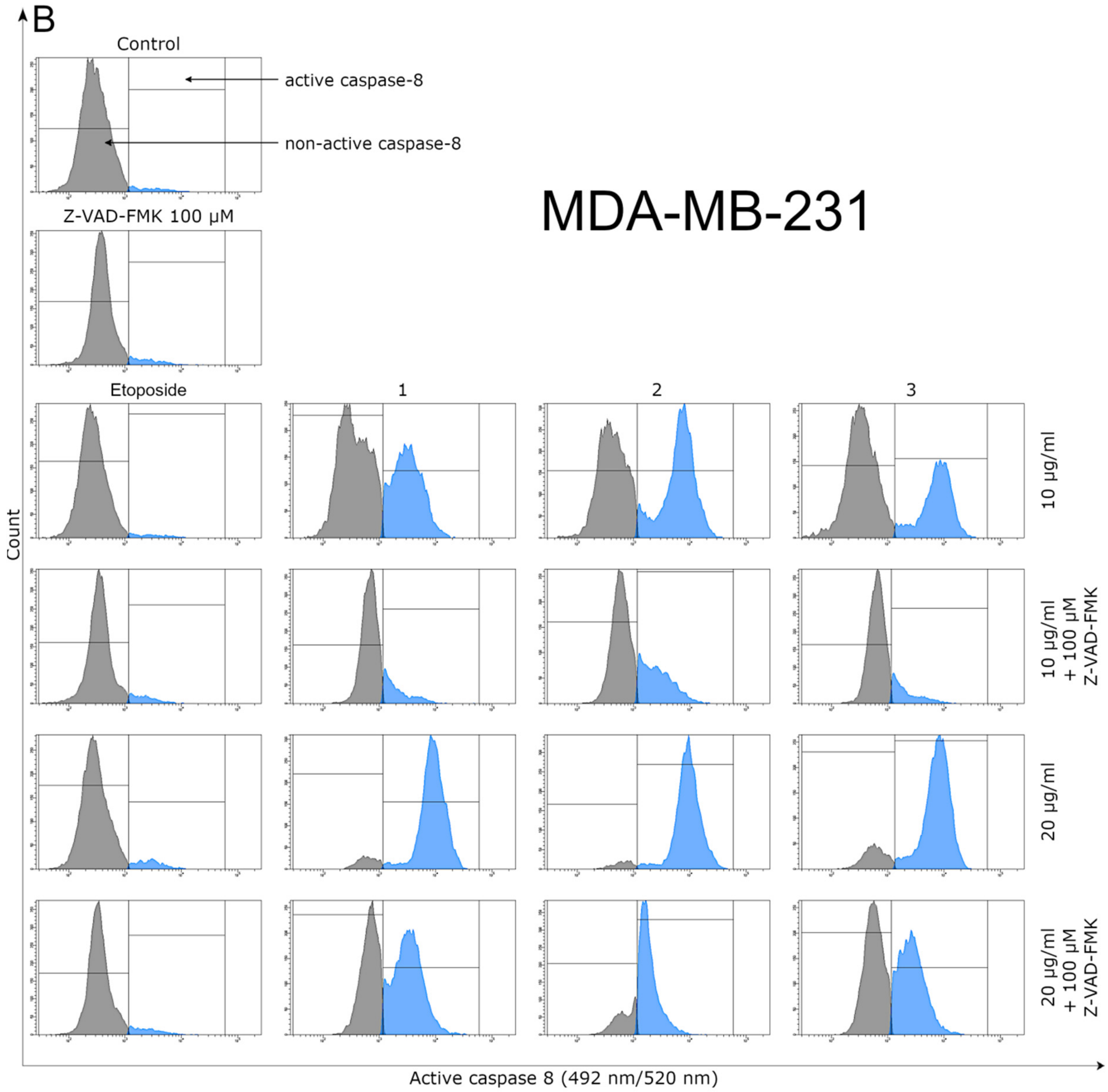
B
MDA-MB-231
Control
active caspase-8
non-active caspase-8
Z-VAD-FMK 100 μM
Etoposide
1
2
3
10 μg/ml
10 μg/ml + 100 μM Z-VAD-FMK
20 μg/ml
20 μg/ml + 100 μM Z-VAD-FMK
Count
Active caspase 8 (492 nm/520 nm)

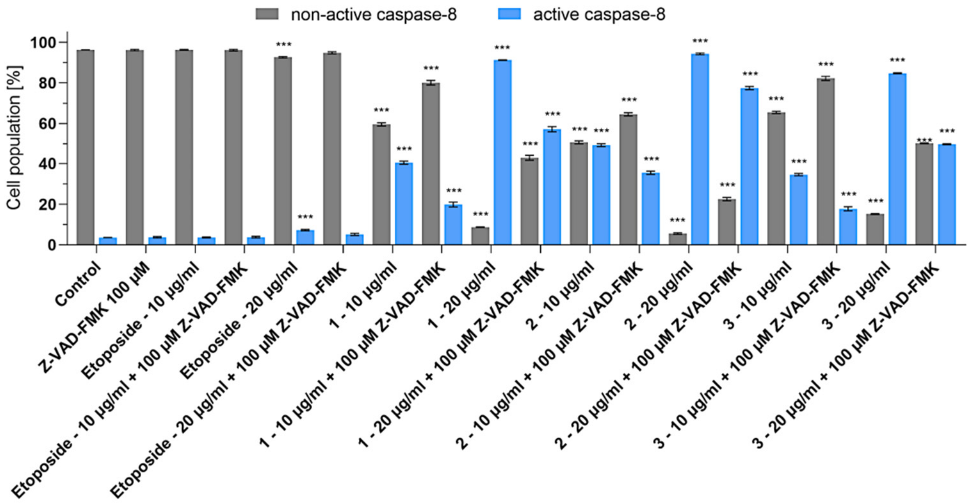
non-active caspase-8
active caspase-8
Cell population [%]
100
80
60
40
20
0
Control
Z-VAD-FMK 100 μM
Etoposide - 10 μg/ml
Etoposide - 10 μg/ml + 100 μM Z-VAD-FMK
Etoposide - 20 μg/ml
Etoposide - 20 μg/ml + 100 μM Z-VAD-FMK
1 - 10 μg/ml
1 - 10 μg/ml + 100 μM Z-VAD-FMK
1 - 20 μg/ml
1 - 20 μg/ml + 100 μM Z-VAD-FMK
2 - 10 μg/ml
2 - 10 μg/ml + 100 μM Z-VAD-FMK
2 - 20 μg/ml
2 - 20 μg/ml + 100 μM Z-VAD-FMK
3 - 10 μg/ml
3 - 10 μg/ml + 100 μM Z-VAD-FMK
3 - 20 μg/ml
3 - 20 μg/ml + 100 μM Z-VAD-FMK

Figure S1. Flow cytometric analysis of MCF-7 (**A**) and MDA-MB-231 (**B**) breast cancer cells treated for 24 h with **1-3** and etoposide for active caspase-8 in the absence or presence of pan-caspase inhibitor (Z-VAD-FMK 100 µM). The compounds were tested at the following concentrations: **1** (10 µg/ml/23.83 µM, 20 µg/ml/47.66 µM), **2** (10 µg/ml/22.75 µM, 20 µg/ml/45.50 µM), **3** (10 µg/ml/23.78 µM, 20 µg/ml/47.56 µM), **etoposide** (10 µg/ml/17 µM, 20 µg/ml/34 µM). Mean percentage values from three independent experiments (n=3) done in duplicate are presented. **p < 0.01 vs. control (Z-VAD-FMK 100 µM) group, ***p < 0.001 vs. control (Z-VAD-FMK 100 µM) group.

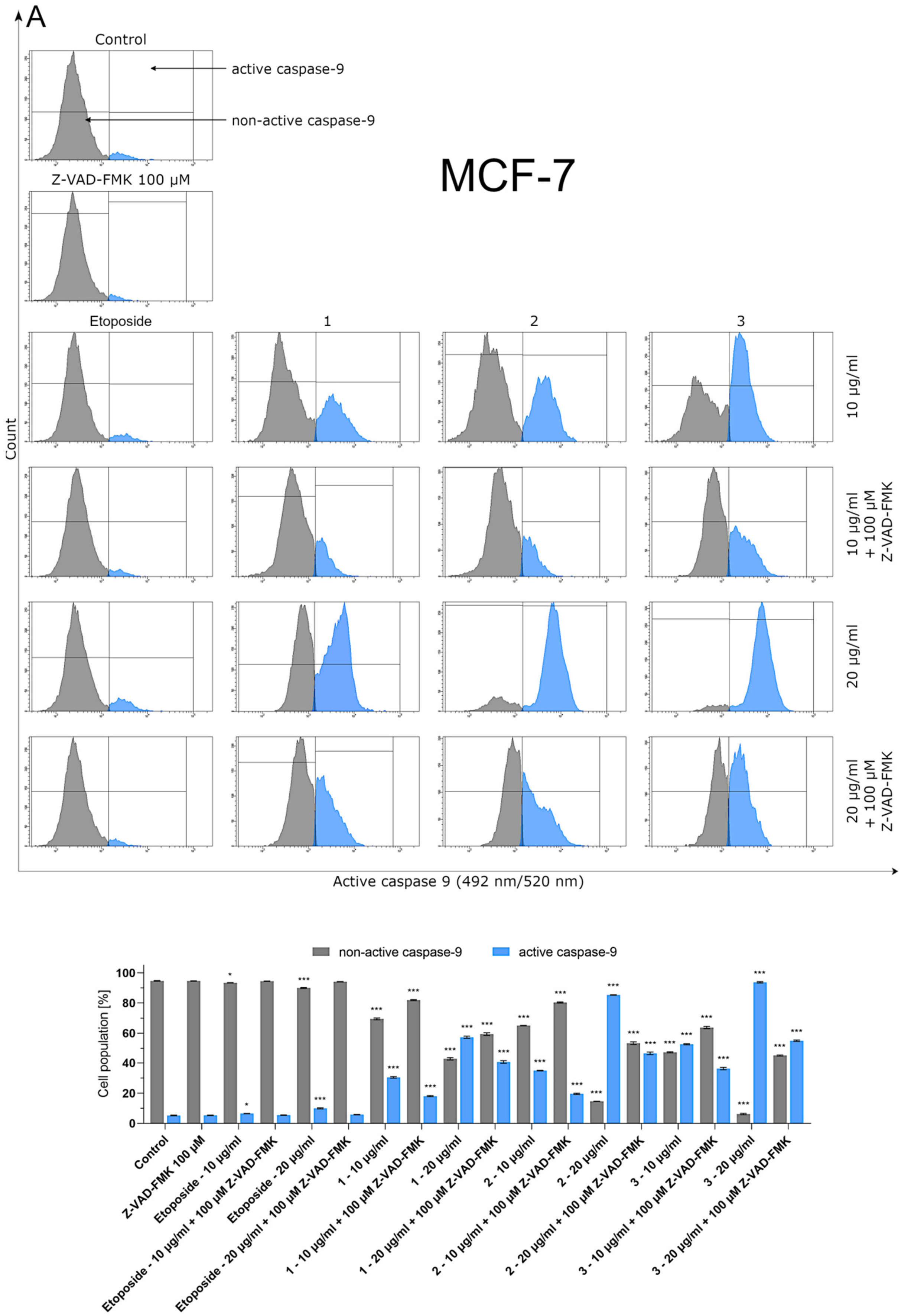
A
Control
active caspase-9
non-active caspase-9
Z-VAD-FMK 100 µM
MCF-7
Etoposide
1
2
3
10 µg/ml
10 µg/ml + 100 µM Z-VAD-FMK
20 µg/ml
20 µg/ml + 100 µM Z-VAD-FMK
Count
Active caspase 9 (492 nm/520 nm)
non-active caspase-9
active caspase-9
Cell population [%]
100
80
60
40
20
0
Control
Z-VAD-FMK 100 µM
Etoposide - 10 µg/ml
Etoposide - 10 µg/ml + 100 µM Z-VAD-FMK
Etoposide - 20 µg/ml
Etoposide - 20 µg/ml + 100 µM Z-VAD-FMK
1 - 10 µg/ml
1 - 10 µg/ml + 100 µM Z-VAD-FMK
1 - 20 µg/ml
1 - 20 µg/ml + 100 µM Z-VAD-FMK
2 - 10 µg/ml
2 - 10 µg/ml + 100 µM Z-VAD-FMK
2 - 20 µg/ml
2 - 20 µg/ml + 100 µM Z-VAD-FMK
3 - 10 µg/ml
3 - 10 µg/ml + 100 µM Z-VAD-FMK
3 - 20 µg/ml
3 - 20 µg/ml + 100 µM Z-VAD-FMK

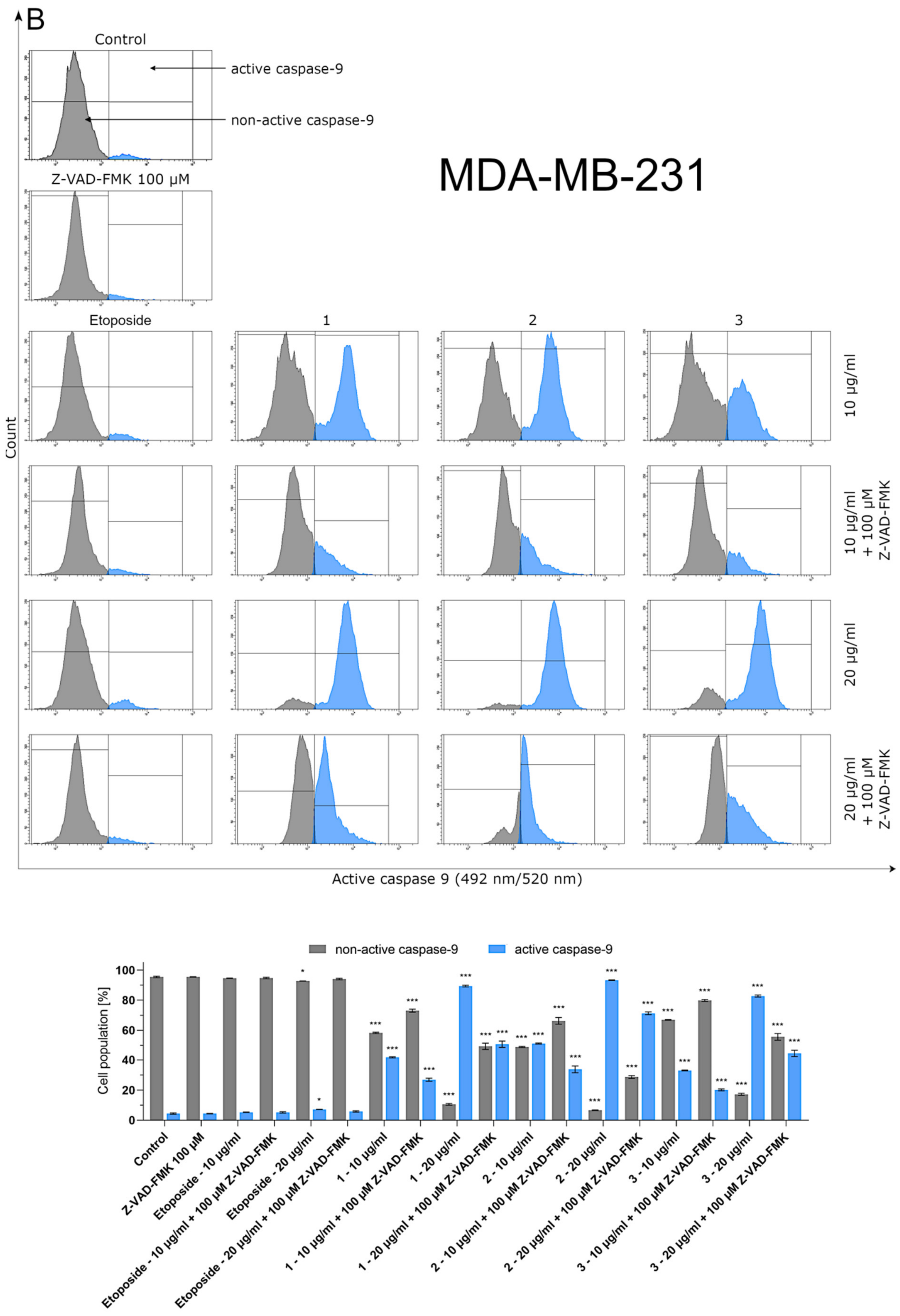
B
MDA-MB-231
Control
active caspase-9
non-active caspase-9
Z-VAD-FMK 100 µM
Etoposide
1
2
3
10 µg/ml
10 µg/ml + 100 µM Z-VAD-FMK
20 µg/ml
20 µg/ml + 100 µM Z-VAD-FMK
Count
Active caspase 9 (492 nm/520 nm)
non-active caspase-9
active caspase-9
Cell population [%]
Control
Z-VAD-FMK 100 µM
Etoposide - 10 µg/ml
Etoposide - 10 µg/ml + 100 µM Z-VAD-FMK
Etoposide - 20 µg/ml
Etoposide - 20 µg/ml + 100 µM Z-VAD-FMK
1 - 10 µg/ml
1 - 10 µg/ml + 100 µM Z-VAD-FMK
1 - 20 µg/ml
1 - 20 µg/ml + 100 µM Z-VAD-FMK
2 - 10 µg/ml
2 - 10 µg/ml + 100 µM Z-VAD-FMK
2 - 20 µg/ml
2 - 20 µg/ml + 100 µM Z-VAD-FMK
3 - 10 µg/ml
3 - 10 µg/ml + 100 µM Z-VAD-FMK
3 - 20 µg/ml
3 - 20 µg/ml + 100 µM Z-VAD-FMK

Figure S2. Flow cytometric analysis of MCF-7 (**A**) and MDA-MB-231 (**B**) breast cancer cells treated for 24 h with **1-3** and etoposide for active caspase-9 in the absence or presence of pan-caspase inhibitor (Z-VAD-FMK 100 μM). The compounds were tested at the following concentrations: **1** (10 μg/ml/23.83 μM, 20 μg/ml/47.66 μM), **2** (10 μg/ml/22.75 μM, 20 μg/ml/45.50 μM), **3** (10 μg/ml/23.78 μM, 20 μg/ml/47.56 μM), **etoposide** (10 μg/ml/17 μM, 20 μg/ml/34 μM). Mean percentage values from three independent experiments (n=3) done in duplicate are presented. **p < 0.01 vs. control (Z-VAD-FMK 100 μM) group, ***p < 0.001 vs. control (Z-VAD-FMK 100 μM) group.
